# Supplementary material for: Monitoring site-specific conformational changes in real-time reveals a misfolding mechanism of the prion protein
Source: eLife. 2019 Jun 24;8:e44698. doi: 10.7554/eLife.44698 (PMC6590988; doi:10.7554/eLife.44698)
Supplement: Supplementary file 1. [file elife-44698-supp1.docx]

| **Protein** | | | **Quantum Yield** | **J(λ) X 10^-13^**  **(M^-1^cm^-1^nm^4^)** | **R_0_ (Å)*** | **FRET**  **Efficiency**  **(measured)** | **FRET efficiency (expected)** |
| --- | --- | --- | --- | --- | --- | --- | --- |
| W144-C153 | | | 0.09 | 8.8 | 23.0 | 0.75 | 0.87 |
| W144-C199 | | | 0.10 | 5.4 | 21.8 | 0.60 | 0.62 |
| W144-C223 | | | 0.10 | 6.4 | 22.3 | 0.09 | 0.06 |
| W197-C169 | TNB | | 0.17 | 6.8 | 24.5 | 0.05 | 0.15 |
|  | DANS | | 0.18 | 8.9 | 25.9 | 0.07 | 0.19 |
| W197-C223 | | TNB | 0.18 | 5.5 | 23.9 | 0.11 | 0.15 |
|  |  | DNP | 0.17 | 20.9 | 29.9 | 0.39 | 0.45 |
|  |  | DANS | 0.18 | 8.7 | 25.8 | 0.13 | 0.22 |

*κ^2^ was assumed to be 2/3 based on time-resolved anisotropy measurements of the two tryptophan residues, W144 and W197 in single Trp-containing mutant variants of the protein and their 1,5-IAEDANS labelled counterparts (Figure 4-figure supplement 4 and Supplementary File 5). R_0_ was calculated using equation 5 in the main text.
