# Supplementary material for: Monitoring site-specific conformational changes in real-time reveals a misfolding mechanism of the prion protein
Source: eLife. 2019 Jun 24;8:e44698. doi: 10.7554/eLife.44698 (PMC6590988; doi:10.7554/eLife.44698)
Supplement: Supplementary file 2. [file elife-44698-supp2.docx]

| **Protein** | **ΔG (kcal mol^-1^)** | **C_m_(M)** | **Rate constant (h^-1^)** |
| --- | --- | --- | --- |
| WT | 4.6±0.2 | 3.82±0.11 | 0.15±0.07 |
| Trp-less | 4.0±0.4 | 3.35±0.09 | 0.08±0.01 |
| W144-C153 | 4.0±0.1 | 3.31±0.05 | 0.16±0.05 |
| W144-C153-TNB | 4.1±0.2 | 3.36±0.03 | 0.18±0.09 |
| W144-C199 | 3.4±0.3 | 2.88±0.08 | 0.24±0.13 |
| W144-C199-TNB | 4.1±0.3 | 3.32±0.05 | 0.77±0.06 |
| W144-C223 | 3.8±0.2 | 3.19±0.03 | 0.14±0.07 |
| W144-C223-TNB | 3.3±0.3 | 2.79±0.13 | 0.61±0.11 |
| W197-C169 | 3.9±0.2 | 3.35±0.19 | 0.36±0.03 |
| W197-C169-TNB | 2.8±0.3 | 3.37±0.04 | 0.69±0.21 |
| W197-C223 | 4.1±0.3 | 3.25±0.12 | 0.15±0.17 |
| W197-C223-TNB | 4.0±0.3 | 2.37±0.04 | 0.78±0.04 |
| W197-C169-DANS | 4.4±0.1(3.2±0.2)^†^ | 3.6±0.1(3.2±0.1)^†^ | 2.40±0.54 |
| W197-C223-DANS | 4.5±0.2 (3.7±0.1)^†^ | 3.7±0.14(3.4±0.2)^†^ | 1.90±0.15 |

*The m value was constrained to 1.23 kcal mol^-1^ M^-1^ for all the moPrP variants, except W197-C169-TNB. The values in brackets are the local stabilities and C_m_ values obtained from the FRET ratio for the DANS-labelled mutant variants shown in Figure 4-figure supplement 5. Error bars are standard deviation of the mean, determined from three independent measurements.
