# Supplementary material for: Monitoring site-specific conformational changes in real-time reveals a misfolding mechanism of the prion protein
Source: eLife. 2019 Jun 24;8:e44698. doi: 10.7554/eLife.44698 (PMC6590988; doi:10.7554/eLife.44698)
Supplement: Supplementary file 3. — The total protein concentration in each case was 100 µM. [file elife-44698-supp3.docx]

| **Protein** | **Rate constant (h^-1^)** |
| --- | --- |
| Trp-less | 0.08±0.01 |
| 1:99 (1 mol%) | 0.08±0.01 |
| 1:50 (2 mol%) | 0.08±0.01 |
| 1:10 (10 mol%) | 0.12±0.01 |
| 1:5 (20 mol%) | 0.12±0.03 |
| W197-C223-TNB | 0.78±0.04 |

*Error bars are standard deviation of the mean, determined from three independent measurements.
