# Supplementary material for: Monitoring site-specific conformational changes in real-time reveals a misfolding mechanism of the prion protein
Source: eLife. 2019 Jun 24;8:e44698. doi: 10.7554/eLife.44698 (PMC6590988; doi:10.7554/eLife.44698)
Supplement: Supplementary file 4. [file elife-44698-supp4.docx]

| **Protein** | **Total protein concentration (µM) & doping ratio** | **k_app_ (h^-1^)** |
| --- | --- | --- |
| W144-C153-TNB | 100, 2 mol% | 0.08±0.02 |
| W197-C223-TNB | 100, 2 mol% | 0.11±0.01 |

*Error bars are standard deviation of the mean, determined from three to five independent measurements. ^†^The apparent rate constant of co-oligomerization monitored by steady-state tryptophan fluorescence anisotropy in Figure 2C.
