# Supplementary material for: Monitoring site-specific conformational changes in real-time reveals a misfolding mechanism of the prion protein
Source: eLife. 2019 Jun 24;8:e44698. doi: 10.7554/eLife.44698 (PMC6590988; doi:10.7554/eLife.44698)
Supplement: Supplementary file 5. [file elife-44698-supp5.docx]

| **Protein** | **Amplitudes** | **Rotational correlation times (ns)** | **r(0) from fit** |
| --- | --- | --- | --- |
| W197 (monomer) | 65% | 0.20 | 0.22 |
|  | 35% | 3.33 |  |
| W197 (oligomer) | 46% | 0.52 | 0.24 |
|  | 54% | 940.00 |  |
| C169-DANS (monomer) | 68% | 0.33 | 0.21 |
|  | 32% | 10.00 |  |
| C223-DANS (monomer) | 64% | 0.40 | 0.24 |
|  | 36% | 6.24 |  |
| C169-DANS (oligomer) | 42.5% | 0.29 | 0.28 |
|  | 57.5% | 940.00 |  |
| C223-DANS (oligomer) | 53% | 0.35 | 0.29 |
|  | 47% | 111.00 |  |
